# Supplementary material for: Interaction estimation of pathogenicity determinant protein βC1 encoded by Cotton leaf curl Multan Betasatellite with Nicotiana benthamiana Nuclear Transport Factor 2
Source: PeerJ. 2022 Nov 15;10:e14281. doi: 10.7717/peerj.14281 (PMC9673767; doi:10.7717/peerj.14281)
Supplement: Supplemental Information 3 [file peerj-10-14281-s003.docx]

| **NCBI Accession No.** | **Predicted Proteins** |
| --- | --- |
| XR_002069373 | PREDICTED: Nicotiana attenuata F-box/kelch-repeat protein SKIP11-like (LOC109235341), transcript variant X3, misc_RNA |
| XM_019401497 | PREDICTED: Nicotiana attenuata MLP-like protein 423 (LOC109235407), Mrna |
| XM_016599582 | PREDICTED: Nicotiana tabacum eukaryotic initiation factor 4A-3-like (LOC107779209), mRNA |
| XR_002069373 | PREDICTED: Nicotiana attenuata F-box/kelch-repeat protein SKIP11-like (LOC109235341), transcript variant X3, misc_RNA |
| XM_019381423 | PREDICTED: Nicotiana attenuata nuclear transport factor 2-like (LOC109217199), mRNA |
| EU849598 | Nicotiana benthamiana subtilisin-like protein mRNA, partial cds |
| XM_019381423 | PREDICTED: Nicotiana attenuata nuclear transport factor 2-like (LOC109217199), mRNA |
| XM_016600895 | PREDICTED: Nicotiana tabacum ferredoxin (LOC107780371), mRNA |
| XM_019381423 | PREDICTED: Nicotiana attenuata nuclear transport factor 2-like (LOC109217199), mRNA |
| XM_016600895 | PREDICTED: Nicotiana tabacum ferredoxin (LOC107780371), mRNA |

Supplementary Table 3: List of ten selected proteins closely related to NTF2.
